# Vigor and Turnover Intention in Nursing Professionals: A Cross‐Sectional Study From Spain

**DOI:** 10.1155/jonm/6886009

**Published:** 2026-09-29

**Authors:** Fernando Díez Ruiz, José Ramón Sánchez Isla, Elene Igoa Iraola, Ángela González Barbadillo, Diana Ochoa Tamayo, Miren Karmele Araujo Díez, Elena Quevedo Torrientes

**Affiliations:** ^1^ Universidad de Deusto, Bilbao, Spain

**Keywords:** Job Demands-Resources model, nursing, retention, turnover intention, vigor, work engagement

## Abstract

**Background:**

This study examines the relationship between work vigor, a core dimension of work engagement, and turnover intention among nursing professionals in Spain, drawing on the Job Demands‐Resources (JD‐R) model.

**Methods:**

A cross‐sectional survey was conducted with 182 nurses from the Basque Country using the Utrecht Work Engagement Scale (UWES‐9) and a three‐item measure of turnover intention.

**Results:**

Data were analyzed through nonparametric statistics, including Spearman’s correlation and group comparisons. Results revealed a significant negative association between vigor and turnover intention (*ρ* = −0.44, *p* < 0.001), indicating that nurses with higher levels of energy and resilience were less likely to consider leaving their jobs. Younger and nontenured professionals showed higher turnover intention scores. These findings suggest that vigor may represent a key psychological resource that is associated with lower withdrawal tendencies in demanding healthcare environments.

**Conclusion:**

The study highlights the need for HR and leadership practices that sustain nurses’ energy and motivation as a potential way to address turnover risk and strengthen workforce stability.

**Implications for Nursing Management:**

Nurse leaders should monitor nurses’ energy and engagement levels, especially among younger and nontenured professionals, and may consider strategies such as supportive leadership, recognition, manageable workloads, mentoring, and career development opportunities to support workforce stability.

## 1. Introduction

Nurse turnover remains one of the most pressing challenges in healthcare systems worldwide. The intention to leave one’s job, understood as the subjective probability of an employee voluntarily exiting their position in the near future, has been consistently linked to adverse outcomes such as staff shortages, decreased quality of care, increased workload for remaining staff, and elevated organizational costs. This issue is especially acute in the nursing profession, where emotional strain, demanding shifts, and limited opportunities for advancement compound the risk of workforce attrition [1, 2].

A broad range of individual and organizational factors have been identified as predictors of turnover intention. Among individual characteristics, age, professional experience, shift work disorders, and emotional exhaustion are prominent. At the organizational level, leadership style, salary satisfaction, social support, and institutional culture play significant roles [3, 4]. Additionally, job instability and interpersonal conflicts have also emerged as strong drivers of disengagement and attrition.

In contrast, organizational commitment has been repeatedly identified as a key protective factor. Employees who feel psychologically connected to their institutions and derive meaning from their work are less likely to consider leaving. Within this framework, work engagement, and more specifically vigor, defined as high levels of energy, resilience, and willingness to invest effort, has shown promise as a critical antecedent of organizational commitment and retention.

In the Spanish healthcare context, work engagement has received growing attention in recent years, particularly among nursing professionals. Previous studies have highlighted the role of engagement in protecting nurses’ wellbeing during highly demanding periods such as the COVID‐19 pandemic [5], as well as the influence of emotional intelligence on engagement levels in healthcare workers [6]. Additionally, integrative reviews conducted in the country have emphasized the relevance of work engagement for nurses’ occupational wellbeing, performance, and retention [7]. However, despite this growing body of research, limited attention has been paid to the specific contribution of the vigor dimension of work engagement in explaining turnover intention.

Despite extensive research on work engagement and turnover intention [2, 8, 9], most studies have treated engagement as a global construct rather than examining its specific dimensions. This study contributes to the literature by examining the specific role of the vigor dimension within work engagement in relation to turnover intention among nursing professionals. This approach allows a more detailed examination of whether the energetic component of work engagement is more strongly associated with turnover intention than the other engagement dimensions. Recent evidence suggests that vigor may be more strongly associated with job continuity than other engagement facets such as dedication or absorption [10, 11]. However, empirical analyses isolating vigor as a distinct variable remain scarce, particularly within the nursing profession, where sustained energy and resilience are critical to coping with emotional and physical demands. Addressing this gap is essential to refine theoretical models of engagement and to inform targeted interventions for workforce stability in healthcare organizations.

The present study aims to examine the relationship between vigor and turnover intention among nursing professionals in the Basque Country (Spain). More specifically, it investigates whether vigor, measured as part of the Utrecht Work Engagement Scale (UWES‐9), can serve as a supportive factor against the intention to leave the profession.

This study hypothesizes that higher levels of vigor are negatively associated with turnover intention. By exploring this relationship, the study seeks to generate evidence‐based insights that may inform organizational strategies to retain nursing talent and enhance the quality of healthcare delivery.

Beyond examining the association between work engagement and turnover intention, this study contributes by examining the specific role of the vigor dimension within the Job Demands‐Resources (JD‐R) framework. This approach allows a more precise understanding of how different dimensions of work engagement relate to turnover intention among nurses. Additionally, the study contributes context‐specific evidence from Spain, where research on engagement in nursing has primarily focused on burnout and general wellbeing rather than on the differential role of vigor in turnover processes.

### 1.1. Theoretical Background

Turnover intention has been widely recognized as a multifaceted phenomenon with significant implications for organizational sustainability and service quality. In the nursing profession, it is particularly problematic, as it directly affects team cohesion, patient care continuity, and institutional costs [2, 12]. This concern is especially acute among younger professionals, who report higher intention to leave due to unmet expectations and limited early‐career support [13]. Existing literature has identified a range of predictors for turnover intention, including emotional exhaustion, job insecurity, interpersonal conflict, and lack of professional development opportunities [1, 4, 14–16].

Among the protective factors that have been explored, organizational commitment stands out as one of the most consistent buffers against turnover [8]. Organizational commitment refers to the emotional and psychological attachment of individuals to their workplace, often acting as a mediator between contextual variables and intention to leave. However, the mechanisms that sustain commitment remain underexplored, especially in high‐pressure environments such as healthcare.

One of the most relevant constructs to emerge in recent years is work engagement, understood as a positive, fulfilling work‐related state of mind characterized by vigor, dedication, and absorption [17]. Engagement has been associated with numerous positive outcomes, including job satisfaction, performance, and retention [18]. Within this model, vigor is considered the foundational dimension, representing high levels of energy, mental resilience, and willingness to invest effort even in the face of challenges.

Recent research suggests that the vigor dimension may show differential associations with organizational outcomes compared with other dimensions of work engagement [19]. Vigor reflects not only a resource‐based disposition but also a motivational force that sustains proactive behavior and emotional endurance. This interpretation is consistent with the JD‐R model, which conceptualizes vigor as a personal resource that can buffer the effects of job strain and enhance adaptive performance over time. In demanding professions such as nursing, characterized by shift work, emotional labor, and physical strain, vigor becomes especially relevant as a protective resource that can mitigate the risks of burnout and disengagement [11].

Furthermore, vigor has been linked to individual traits such as empathy, emotional intelligence, and professional purpose [10], which are especially prominent in care‐based professions. This multidimensional capacity enables nurses to maintain effectiveness in emotionally complex situations and is therefore hypothesized to be inversely related to the desire to leave the organization. To operationalize vigor, validated scales such as the UWES‐9 have proven effective in capturing its energizing dimension across sectors, including healthcare [20, 21].

Despite its theoretical importance, empirical evidence on the specific relationship between vigor and turnover intention in nurses remains limited. Most existing studies consider work engagement as a whole, without disentangling the specific contribution of each of its components. Yet, internal turnover rates among newly licensed nurses remain high, indicating the urgency of identifying protective psychological factors early in the career cycle [22].

Within the framework of the JD‐R model [23], vigor is understood as a core psychological resource that not only mitigates the impact of job demands but also reinforces sustained motivation and adaptive functioning. Vigor plays a dual role in this model: It contributes directly to positive work outcomes and indirectly protects against strain and disengagement. As highlighted by recent meta‐analyses [24, 25], vigor shows the highest predictive validity in reducing turnover intention when compared to other engagement dimensions, notably in emotionally demanding professions. These insights reinforce its utility as an interpretative framework for the present study and justify its use in both hypothesis formulation and data analysis.

By examining the vigor dimension separately within the broader work engagement framework, this study contributes to a more nuanced understanding of how psychological energy and resilience can serve as strategic levers in organizational retention efforts. Proactive leadership strategies aimed at enhancing engagement have been shown to positively impact these outcomes [26]. The findings may offer valuable insights for human resource management and leadership practices in healthcare institutions. Given the influence of unit‐level factors on turnover, as highlighted in recent studies [27], the results could inform targeted interventions in long‐term care settings.

While work engagement is traditionally conceptualized as a multidimensional construct comprising vigor, dedication, and absorption [17], focusing on vigor is theoretically justified. First, vigor represents the energetic and motivational core of engagement, reflecting the availability of physical and mental resources that sustain performance under pressure [19]. Unlike dedication and absorption, vigor directly reflects the availability of energetic resources necessary to sustain care‐related performance under chronic strain. This distinction is particularly relevant in nursing contexts characterized by emotional overload, shift work, and sustained interpersonal demands. Second, empirical research has shown that vigor exhibits the highest predictive validity for positive work outcomes, including job satisfaction, commitment, and reduced turnover, especially in high‐demand professions such as nursing [10, 11, 25]. Third, analyzing vigor separately allows for a finer understanding of the mechanisms through which engagement influences retention, consistent with calls for more nuanced analyses within the JD‐R framework [28]. Therefore, the present study focuses on the vigor dimension as a theoretically relevant component of work engagement that may show particular relevance for turnover intention.

### 1.2. Conceptual Model and Hypotheses

Grounded in the JD‐R model [23], this study conceptualizes vigor as a personal resource that enhances adaptive functioning and reduces withdrawal intentions under demanding work conditions. In this framework, vigor represents a key motivational factor capable of buffering the negative effects of job demands on nurses’ intention to leave. Turnover intention, in turn, is viewed as a behavioral outcome reflecting reduced organizational attachment and future withdrawal tendencies.

Based on previous empirical evidence [10, 11, 19], the following hypotheses are proposed:

 H1: Work vigor is negatively associated with turnover intention among nursing professionals.
 H2: Nurses with higher job stability (permanent contracts) will report lower turnover intention than those without tenure.
 H3: Age and professional experience will be negatively related to turnover intention.

These relationships are represented in Figure 1, which depicts vigor as a key predictor of turnover intention, including job stability, age, and professional experience as additional predictors.

**FIGURE 1:**
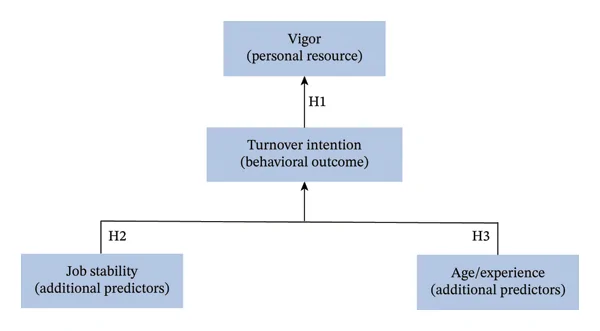
Proposed hypotheses for this study.

## 2. Methodology

### 2.1. Research Design

This study employed a cross‐sectional, observational design to examine the relationship between work engagement, specifically the vigor dimension, and turnover intention among active nursing professionals. This design is suitable for identifying associations between psychological variables and behavioral intentions at a specific point in time, allowing for the exploration of correlational patterns without manipulating variables.

### 2.2. Participants and Sampling

A convenience sample of 182 active registered nurses working in the Basque Country (Spain) was recruited. Participants were invited to participate through nursing professional associations, academic networks, and collaborating clinical institutions. Inclusion criteria required participants to be currently employed in healthcare settings and actively practicing as nurses.

Sample size estimation was conducted based on an *α* level of 0.05, a statistical power of 80% (*β* = 0.2), and a minimum effect size of 0.1. These parameters yielded a required sample of 180 participants for reliable estimation of means and a minimum of six per subgroup for intergroup comparisons. The final sample met these requirements.

### 2.3. Data Collection Procedures

Data were collected using an online questionnaire administered via the Qualtrics platform. A formal invitation was distributed with an explanatory note, informed consent form, and a secure link to the questionnaire. Participation was anonymous and voluntary. The study received ethical approval from the Deusto Research Ethics Committee on December 19, 2023 (approval code: ETK‐44/23‐24).

### 2.4. Instruments and Variables

The survey consisted of three sections:

1. Turnover Intention: Turnover intention was measured using three items conceptually grounded in previous organizational turnover research, particularly the Mobley turnover framework and related occupational stress literature [29–31]. The items assessed the frequency of thoughts about leaving the current organization and the perceived likelihood of leaving the current job in the short term (1 year) and medium term (5 years). The items were adapted to the nursing context and administered in Spanish. Responses were collected using a 7‐point Likert scale (1 = never/totally unlikely to 7 = always/totally likely). A composite turnover intention score was calculated as the mean of the three items. For descriptive purposes only, scores ≥ 4 were used to identify participants reporting relatively elevated turnover intention. This threshold was not treated as a validated clinical or diagnostic cutoff, but as a practical reference point based on the midpoint of the response scale.
2. Work Engagement‐Vigor: Measured using the Spanish short version of the UWES‐9, which includes three subscales: vigor, dedication, and absorption. Each item was rated on a 7‐point Likert scale (0 = never to 6 = always). The vigor subscale score was analyzed separately and compared with the overall engagement score. The vigor subscale demonstrated satisfactory internal consistency in the present sample (Cronbach’s *α* = 0.896). The turnover intention scale also demonstrated acceptable internal consistency (Cronbach’s *α* = 0.731).
3. Although vigor is one of the three dimensions of the UWES‐9, the present study examined this dimension separately because previous theoretical and empirical research has suggested that it may show stronger associations with retention‐related outcomes than dedication or absorption. The findings should therefore be interpreted as referring to a specific dimension of work engagement rather than as evidence of an independent psychological construct.
4. Sociodemographic and Professional Variables: Collected using an ad hoc questionnaire, including age, gender identity, nursing qualifications (ATS, DUE, BSc), postgraduate education, nursing specialty, sector (public or private), job stability (tenure), years of professional experience, work area (e.g., emergency, primary care, surgery), managerial responsibilities, and current work shift.

### 2.5. Statistical Analysis

Data were analyzed using SPSS Version 25.0. Descriptive statistics were used to summarize sample characteristics and variable distributions. The Kolmogorov–Smirnov test revealed non‐normal distributions for both engagement and turnover intention scores, as well as for their subcomponents. Consequently, nonparametric statistical tests were applied:

• Spearman’s Rho for correlational analysis.
• Mann–Whitney *U* test for comparisons between two independent groups.
• Kruskal–Wallis *H* test for comparisons across multiple groups.

Effect sizes were calculated using epsilon‐squared (*ε* ^2^) and rank‐biserial correlation (*r*) where appropriate. The level of statistical significance was set at *p* < 0.05. Despite deviations from normality in some study variables, multiple linear regression was conducted because residual diagnostics confirmed acceptable normality, homoscedasticity, and linearity assumptions, supporting the robustness of the model estimates.

## 3. Results

A total of 182 nurses participated in the study. The mean age was 42.24 years (SD = 11.47), with a range between 23 and 67. Most participants identified as women (79.1%), with men representing 19.8% and 1.1% identifying as nonbinary. Educational qualifications and work experience were diverse, and 60.4% were employed in the public sector. Additional sociodemographic characteristics are summarized in Table 1.

**TABLE 1:** Sociodemographic characteristics.

Variable	Value
Age (M ± SD)	42.24 ± 11.47
Gender—women	79.1% (*n* = 144)
Gender—men	19.8% (*n* = 36)
Tenure—with position	62.1% (*n* = 113)
Tenure—without position	37.9% (*n* = 69)

*Note:* Age is reported as mean and standard deviation (M ± SD). Tenure refers to having a permanent position. Percentages are rounded to one decimal place. Sample characteristics (*N* = 182).

### 3.1. Turnover Intention

The mean turnover intention score was 3.42 (SD = 1.58), with item‐level means ranging from 2.87 to 3.91. Table 2 displays item statistics and the composite ‐score.

**TABLE 2:** Descriptive statistics for turnover intention items (*N* = 182).

Item	Mean	Median	SD	Observed range	
				Min	Max
Overall turnover intention (composite score)	3.42	3.33	1.58	1	6.67
Desire to leave current organization	3.48	3.00	2.01	1	7
Likelihood of leaving within the next year	2.87	2.00	2.27	1	7
Likelihood of leaving within the next 5 years	3.91	4.00	2.32	1	7

*Note:* All items were rated on a 7‐point Likert scale (1 = never/totally unlikely, 7 = always/totally likely). The composite score represents the average of the three individual items.

A multiple linear regression analysis was conducted to predict turnover intention based on vigor, age, professional experience, and possession of a specialist qualification in health sciences. The results indicated that the model explained a significant proportion of the variance, adjusted *R* ^2^ = 0.313, *F*(4, 176) = 17.48, *p* < 0.001.

The analysis of the standardized coefficients revealed that vigor was the predictor with the strongest negative effect on turnover intention (*β* = −0.536, *p* < 0.001). Likewise, age (*β* = −0.355, *p* = 0.014) and holding a nursing specialist qualification (*β* = −0.105, *p* = 0.047) were also significant predictors and were inversely associated with the dependent variable. In contrast, professional experience showed a significant positive relationship with turnover intention (*β* = 0.309, *p* = 0.046). Collinearity diagnostics indicated relatively high collinearity between age (tolerance = 0.186, VIF = 5.368) and professional experience (tolerance = 0.161, VIF = 6.215). These values suggest substantial overlap between the two predictors, which may have affected the stability of the regression estimates and contributed to the positive coefficient observed for professional experience in the multivariate model.

### 3.2. Work Engagement and Vigor

The mean UWES‐9 global score was 4.09 (SD = 1.17), with vigor scoring lower than dedication and absorption (*M* = 3.84, SD = 1.33). Table 3 shows the distribution of engagement dimensions. Normality tests (Kolmogorov–Smirnov) indicated significant deviation across all variables (*p* < 0.05), justifying the use of nonparametric analyses.

**TABLE 3:** Descriptive statistics for work engagement (UWES‐9) and subdimensions (*N* = 182).

Dimension	Mean	Median	SD	Observed range	
				Min	Max
Total work engagement (UWES‐9)	4.09	4.22	1.17	0	6
Vigor	3.84	4.00	1.33	0	6
Dedication	4.35	4.50	1.24	0	6
Absorption	4.09	4.00	1.17	0	6

*Note:* Scores range from 0 (never) to 6 (always). The UWES‐9 evaluates work engagement through three subscales: Vigor, Dedication, and Absorption.

### 3.3. Correlational Analysis

Spearman’s correlations revealed moderate, negative, and statistically significant associations between turnover intention and all engagement dimensions. The vigor dimension showed the strongest negative correlation (*ρ* = −0.444, *p* < 0.001), surpassing both dedication (*ρ* = −0.421) and absorption (*ρ* = −0.291). As illustrated in Figure 2, the scatterplot visually confirms this inverse association, showing that higher vigor scores tend to correspond with lower turnover intention scores across participants (see Table 4 and Figure 2).

**FIGURE 2:**
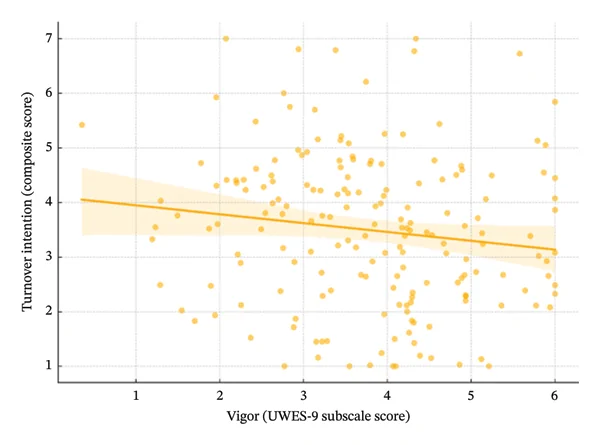
Relationship between vigor and turnover intention. *Note:* Scatterplot showing the negative association between vigor (measured with the UWES‐9 subscale) and turnover intention (composite score from three Likert items). Higher levels of vigor are associated with lower intention to leave.

**TABLE 4:** Correlation between turnover intention and work engagement dimensions (*N* = 182).

Dimension	Work engagement	Work engagement: Vigor	Work engagement: Dedication	Work engagement: Absorption
Turnover intention	*ρ* = −0.414 ^∗∗^ *p* < 0.001	*ρ* = −0.444 ^∗∗^ *p* < 0.001	*ρ* = −0.421 ^∗∗^ *p* < 0.001	*ρ* = −0.291 ^∗∗^ *p* < 0.001
How often do you feel like leaving your job in this organization?	*ρ* = −0.543 ^∗∗^ *p* < 0.001	*ρ* = −0.572 ^∗∗^ *p* < 0.001	*ρ* = −0.566 ^∗∗^ *p* < 0.001	*ρ* = −0.370 ^∗∗^ *p* < 0.001
What is the probability that you will leave your current job within the next year?	*ρ* = −0.279 ^∗∗^ *p* < 0.001	*ρ* = −0.296 ^∗∗^ *p* < 0.001	*ρ* = −0.269 ^∗∗^ *p* < 0.001	*ρ* = −0.220 ^∗∗^ *p* = 0.003
What is the probability that you will change jobs within the next 5 years?	*ρ* = −0.151 ^∗^ *p* = 0.042	*ρ* = −0.165 ^∗^ *p* = 0.026	*ρ* = −0.157 ^∗^ *p* = 0.035	*ρ* = −0.101 *p* = 0.173

*Note: ρ*, Spearman’s rho.

^∗^ *p* < 0.05.

^∗∗^ *p* < 0.01.

### 3.4. Group Comparisons

Age was negatively correlated with the desire to leave the organization (*ρ* = −0.157, *p* = 0.034) and with long‐term turnover intention (*ρ* = −0.169, *p* = 0.022).

Years of experience significantly affected long‐term turnover intention (*H* = 15.83, *p* < 0.001, *ε* ^2^ = 0.027), with less experienced nurses showing higher scores. Post hoc tests confirmed statistically significant differences across experience levels.

Job stability (tenure status) had a significant effect: Nurses without permanent positions reported higher turnover intention (*M* = 4.57) than those with tenure (*M* = 3.53; *U* = 2865, *Z* = −2.77, *p* = 0.006, *r* = −0.16).

## 4. Discussion

This study aimed to examine the relationship between work engagement, particularly the vigor dimension, and turnover intention among nursing professionals. The findings support the initial hypothesis: Higher levels of vigor are significantly associated with lower levels of turnover intention, even more strongly than the global engagement score or other dimensions such as dedication and absorption.

One of the main contributions of this research is showing that the vigor dimension exhibited the strongest association with lower turnover intention among the work engagement dimensions examined. Vigor, characterized by energy, mental resilience, and persistence in the face of challenges, appears to be especially critical in nursing contexts. These results align with Shirom’s [19] conceptualization of vigor as an affective resource that supports long‐term occupational health and motivation. Similar findings were reported by Bakker et al. [18] and Wei et al. [11], who linked vigor to enhanced retention and job satisfaction in high‐demand professions. Moreover, the organizational consequences of turnover, including increased recruitment costs, training investment losses, and disrupted patient care, underscore the strategic importance of proactively fostering vigor in healthcare settings.

These findings are also consistent with previous research conducted in Spain on work engagement among healthcare professionals. For example, Allande‐Cussó et al. [5] reported that engagement acted as a protective psychological resource for nurses during the COVID‐19 pandemic, despite the extreme emotional and organizational pressures experienced in healthcare settings. Similarly, Pérez‐Fuentes et al. [6] highlighted the importance of emotional intelligence in sustaining engagement among nursing professionals, reinforcing the idea that psychological and emotional resources play a central role in occupational wellbeing and retention. Furthermore, the integrative review developed by García‐Sierra et al. [7] emphasized that work engagement constitutes a critical factor for professional sustainability and quality of care in nursing contexts. The present study extends this body of Spanish literature by suggesting that vigor, specifically, may represent the most relevant energetic component associated with lower turnover intention.

However, the buffering role of vigor should not be interpreted in isolation from structural working conditions. Emerging debates in occupational health psychology warn against over‐individualizing engagement, as highly engaged nurses may still experience turnover when organizational demands chronically exceed available institutional resources.

In this regard, healthcare organizations should avoid framing vigor exclusively as an individual responsibility. Although personal psychological resources are important, sustained retention also depends on structural conditions such as staffing adequacy, workload distribution, leadership quality, and organizational justice. Otherwise, there is a risk of promoting resilience‐oriented narratives while leaving systemic organizational stressors unaddressed.

The present results confirm that age was inversely associated with turnover intention, whereas professional experience showed a more complex relationship in the multivariate model. Although previous research generally associates greater professional experience with lower turnover intention, the positive association observed in the regression model should be interpreted cautiously. Given the relatively high collinearity between age and professional experience, this finding may reflect a suppression effect and should be interpreted with caution, as the stability of the regression estimates may have been affected by the overlap between these predictors. An alternative explanation is that more experienced nurses may accumulate occupational strain or develop changing career expectations over time, which could increase their intention to leave under certain organizational conditions. Therefore, future studies should further explore these relationships using longitudinal designs and by incorporating additional organizational variables. Furthermore, job stability, operationalized as holding a permanent position, was also significantly associated with reduced turnover intention, corroborating previous research emphasizing the importance of perceived security and long‐term career prospects in staff retention [4, 32].

Importantly, while dedication and absorption also showed negative correlations with turnover intention, their effect sizes were smaller than that of vigor. This suggests that the energetic and motivational foundation of engagement plays a more decisive role than affective attachment or cognitive immersion alone. From a theoretical perspective, these findings suggest that the vigor dimension may play a particularly relevant role within the broader work engagement construct when examining turnover intention.

From the perspective of the JD‐R model, these findings suggest that vigor may be associated with a motivational process linking personal psychological resources with retention‐related outcomes. In this framework, turnover intention can be interpreted as a form of withdrawal cognition emerging when job demands chronically exceed available resources. Consequently, nurses with higher levels of vigor may report greater adaptive functioning and organizational attachment despite exposure to emotionally demanding work environments. This interpretation reinforces the idea that retention is not exclusively dependent on structural employment conditions, but also on the balance between occupational demands and the psychological resources available to cope with them.

These insights have practical implications: Organizations may consider interventions aimed at supporting vigor among nursing staff, such as recognition programs, manageable workloads, supportive leadership, and professional development opportunities. Such initiatives may contribute to both retention and care quality, as energetic and engaged nurses are more likely to perform well and remain in their roles.

These findings are consistent with Brewer et al. [33] and Wu et al. [9], suggesting that more experienced nurses are less likely to consider leaving, possibly due to increased professional confidence, organizational integration, or more realistic job expectations.

## 5. Practical Implications and Recommendations

The results of this study offer relevant implications for nursing leadership and human resource management in healthcare organizations. Recognizing vigor as a central driver of retention, institutions may consider implementing strategies that actively support the energy, motivation, and emotional resilience of nursing professionals.

First, nursing leaders at all management levels should cultivate environments that reinforce daily motivation. This can include peer support structures, participatory decision‐making, and access to mentoring or coaching. Managers should also be trained to recognize signs of energy depletion and respond proactively to prevent burnout.

Second, organizational policies should promote career stability and development, especially for younger nurses or those in the early stages of their professional careers. Providing access to permanent contracts, advanced training programs, and clear promotion paths may reinforce the perception of long‐term commitment between the organization and the professional.

Third, employee recognition and wellness programs can be powerful tools to reinforce vigor. Flexible scheduling, emotional support resources, and acknowledgment of effort in demanding conditions may help energize staff and reduce the intention to leave.

Finally, the data suggest that targeted retention strategies should prioritize professionals with less than 10 years of experience or without tenure, as they are more likely to consider exiting the profession in the medium term. Tailoring interventions to this at‐risk segment could lead to measurable improvements in retention rates and workforce continuity.

These measures may help address turnover risk and can also enhance institutional performance and care quality, ensuring that engaged and resilient professionals remain at the core of healthcare delivery.

## 6. Conclusion

This study highlights the multidimensional nature of turnover intention among nursing professionals and reinforces the role of psychological engagement, particularly vigor, as a key protective factor. The findings suggest that vigor, more than dedication or absorption, is the dimension most strongly associated with the decision to remain in or leave the profession.

By focusing on the vigor dimension of work engagement, this research adds depth to our understanding of how energy, resilience, and intrinsic motivation are associated with retention. These insights are especially relevant in healthcare environments characterized by emotional strain and staff shortages.

The results align with the initial objectives of the study: To examine whether vigor is associated with lower turnover intention in a demanding occupational context. The consistent, statistically significant associations observed suggest that supporting vigor among nurses may contribute to reducing attrition risk and enhancing organizational sustainability.

Going forward, efforts to address turnover may benefit from interventions that foster and sustain high levels of vigor, notably among early‐career professionals and those without permanent contracts. By doing so, such organizational efforts may support staff wellbeing and contribute to the quality and continuity of patient care.

### 6.1. Limitations and Future Research Directions

While this study provides valuable insights into the relationship between vigor and turnover intention among nursing professionals, several limitations should be acknowledged.

First, the cross‐sectional design prevents establishing causal relationships between the variables studied. Although significant associations were found, the directionality of the relationships remains speculative. Longitudinal studies are recommended to explore how changes in vigor over time may predict turnover decisions more accurately.

Second, the study employed a convenience sampling method, which may limit the generalizability of the results. Although the sample size was sufficient for statistical analysis, it was restricted to nurses in the Basque Country, potentially excluding variability present in other regions, healthcare systems, or organizational settings. In addition, the recruitment strategy may have introduced self‐selection bias, as nurses with stronger opinions about their work conditions, engagement, or intention to leave may have been more likely to participate. Replicating this study across diverse geographic and institutional contexts using probabilistic or stratified sampling strategies would enhance external validity.

Third, although vigor was analyzed as a specific dimension of work engagement, the study did not include other relevant psychological or organizational variables, such as stress, emotional exhaustion, staffing levels, workload, leadership style, team climate, or perceived organizational justice. The absence of these variables limits the ability to determine whether the observed associations between vigor and turnover intention are partly explained by broader working conditions. Future research should incorporate more comprehensive models, such as the JD‐R framework, including both individual‐ and organizational‐level variables.

Lastly, the self‐reported nature of the data may be subject to social desirability or response bias. Because all variables were collected using self‐report measures at a single time point, common method variance cannot be ruled out. Complementing survey‐based data with qualitative methods (e.g., interviews or focus groups) could offer richer insights into how vigor is experienced and expressed in clinical practice.

Future studies might also explore interventions specifically designed to enhance vigor, assessing their impact on retention, job satisfaction, and patient outcomes. Such applied research could guide the development of evidence‐based human resource strategies tailored to the healthcare sector.

## Author Contributions

Fernando Díez Ruiz, José Ramón Sánchez Isla, Elene Igoa Iraola, Ángela González Barbadillo, and Diana Ochoa Tamayo contributed to the study conception and design. Ángela González Barbadillo, Diana Ochoa Tamayo, and José Ramón Sánchez Isla contributed to data collection and analysis. Fernando Díez Ruiz and Elene Igoa Iraola contributed to manuscript drafting. Elena Quevedo Torrientes and Miren Karmele Araujo Díez contributed to critical revision.

## Funding

This research received no specific grant from any funding agency in the public, commercial, or not‐for‐profit sectors.

## Disclosure

All authors approved the final version of the manuscript.

## Conflicts of Interest

The authors declare no conflicts of interest.

## Data Availability

The data that support the findings of this study are available from the corresponding author upon reasonable request.
